# The Neutrophil Nucleus: An Important Influence on Neutrophil Migration and Function

**DOI:** 10.3389/fimmu.2018.02867

**Published:** 2018-12-04

**Authors:** Harriet R. Manley, Maria Cristina Keightley, Graham J. Lieschke

**Affiliations:** Australian Regenerative Medicine Institute, Monash University, Clayton, VIC, Australia

**Keywords:** neutrophils, nucleus, migration, neutrophil extracellular traps, NETs, leukocytes, lamins, lamin B receptor

## Abstract

Neutrophil nuclear morphology has historically been used in haematology for neutrophil identification and characterisation, but its exact role in neutrophil function has remained enigmatic. During maturation, segmentation of the neutrophil nucleus into its mature, multi-lobulated shape is accompanied by distinct changes in nuclear envelope composition, resulting in a unique nucleus that is believed to be imbued with extraordinary nuclear flexibility. As a rate-limiting factor for cell migration, nuclear morphology and biomechanics are particularly important in the context of neutrophil migration during immune responses. Being an extremely plastic and fast migrating cell type, it is to be expected that neutrophils have an especially deformable nucleus. However, many questions still surround the dynamic capacities of the neutrophil nucleus, and which nuclear and cytoskeletal elements determine these dynamics. The biomechanics of the neutrophil nucleus should also be considered for their influences on the production of neutrophil extracellular traps (NETs), given this process sees the release of chromatin “nets” from nucleoplasm to extracellular space. Although past studies have investigated neutrophil nuclear composition and shape, in a new era of more sophisticated biomechanical and genetic techniques, 3D migration studies, and higher resolution microscopy we now have the ability to further investigate and understand neutrophil nuclear plasticity at an unprecedented level. This review addresses what is currently understood about neutrophil nuclear structure and its role in migration and the release of NETs, whilst highlighting open questions surrounding neutrophil nuclear dynamics.

## Introduction

The nucleus has long been considered the cell's control centre, housing genetic material and providing a biochemical factory for DNA replication and RNA synthesis. Being the largest organelle and up to ten times more rigid than the cytoplasm, the nucleus also exerts significant influence on cellular biomechanics (1, 2). Albeit large, the nucleus is not a static organelle; rather it is itself capable of propagating intracellular forces (3) and dynamically changing its shape and integrity (4). Biomechanical roles for the nucleus and its nuclear envelope have been identified during several cellular processes including cell division (5, 6), migration (3, 7), development, and tumourigenesis (4, 8). However, gaps remain in our understanding of how nuclear plasticity specifically impacts cellular flexibility and motility—in particular, that of cancer cells, stem cells, and immune cells like neutrophils.

As the first leukocyte responders of the innate immune system, neutrophils exhibit a unique collection of migratory capabilities. These include high velocity, high deformability, and diverse forms of migration, such as transmigration and reverse migration (9–11). Given that nuclei can transmit traction force through cells as they migrate (3) and nuclear deformability limits migratory speed (12), it can be hypothesised that the nucleus is a key determinant of neutrophil migration. Additionally, neutrophils release neutrophil extracellular traps (NETs) (13). The process of NET formation, often termed NETosis, requires chromatin release and extensive nuclear remodelling, yet it is a process that has not been well-characterised mechanically. In light of new and emerging biological technologies, we are now in a position to examine the impact of nuclear dynamics on neutrophil function, including migration and NETosis.

Neutrophils possess distinctive multi-lobulated nuclei and a particular nuclear envelope protein composition (14). The functional capabilities of neutrophils that are impacted by their nuclear shape, composition and plasticity are fundamental to understanding their cellular biology. As an exhibitor of extreme nuclear plasticity, the neutrophil nucleus also sheds light on the broader nuclear biomechanics. Neutrophils provide a unique cellular model for experimentally modulating a nucleus and demonstrating how specific nuclear components impact nuclear shape, and enhance flexibility and dynamics. This review firstly summarises nuclear biomechanics, and what is known about neutrophil nuclear structure and its influence on neutrophil maturation and migration. Secondly, it presents hypotheses for how the nucleus contributes to the unique plasticity and migratory ability of neutrophils. Thirdly, the neutrophil nucleus is discussed in relation to NET release, and how nuclear mechanisms underpinning NETosis may lead to a greater understanding of this phenomenon.

### Nuclear Biomechanics and the Nuclear Envelope

The nucleus, its nuclear envelope, and the surrounding cytoskeletal network contribute to and receive biomechanical forces that collectively determine nuclear morphology and location (3, 15). The interplay of these forces depends upon the cell type and its activity, with nuclear plasticity being the cumulative result of summed compressive, stretching, and shear stress forces. Greater resistance from the cytoskeleton sees the nucleus behave as a “protective shell,” whereas greater resistance from the nucleus can transduce forces to the extracellular matrix via the cytoskeleton, driving cellular movement [(3, 16), reviewed in (17)]. *In vitro* tests exist to measure factors of nuclear biophysics: including nuclear deformation (flexibility/rigidity, changes in nuclear shape) and compression (fragility/resistance to pressure, changes in nuclear volume). However, a recent cell migration study demonstrated the important influence of 3-D environments on cellular and nuclear behaviour, with the nucleus an absolute requirement for 3D migration but not for migration in 1-D or 2-D contexts (18). To determine nuclear dynamics during complex cell movements in complex 3-D environments, more sophisticated *ex vivo* and *in vivo* techniques are required, particularly if the aim is to elucidate how nuclear envelope components affect these dynamics. The development of new mechanobiological methods (19), animal models (20), microfluidic devices (4, 21), and higher resolution imaging techniques (22–24) equip the field to answer such scientific questions.

**Figure 1:**
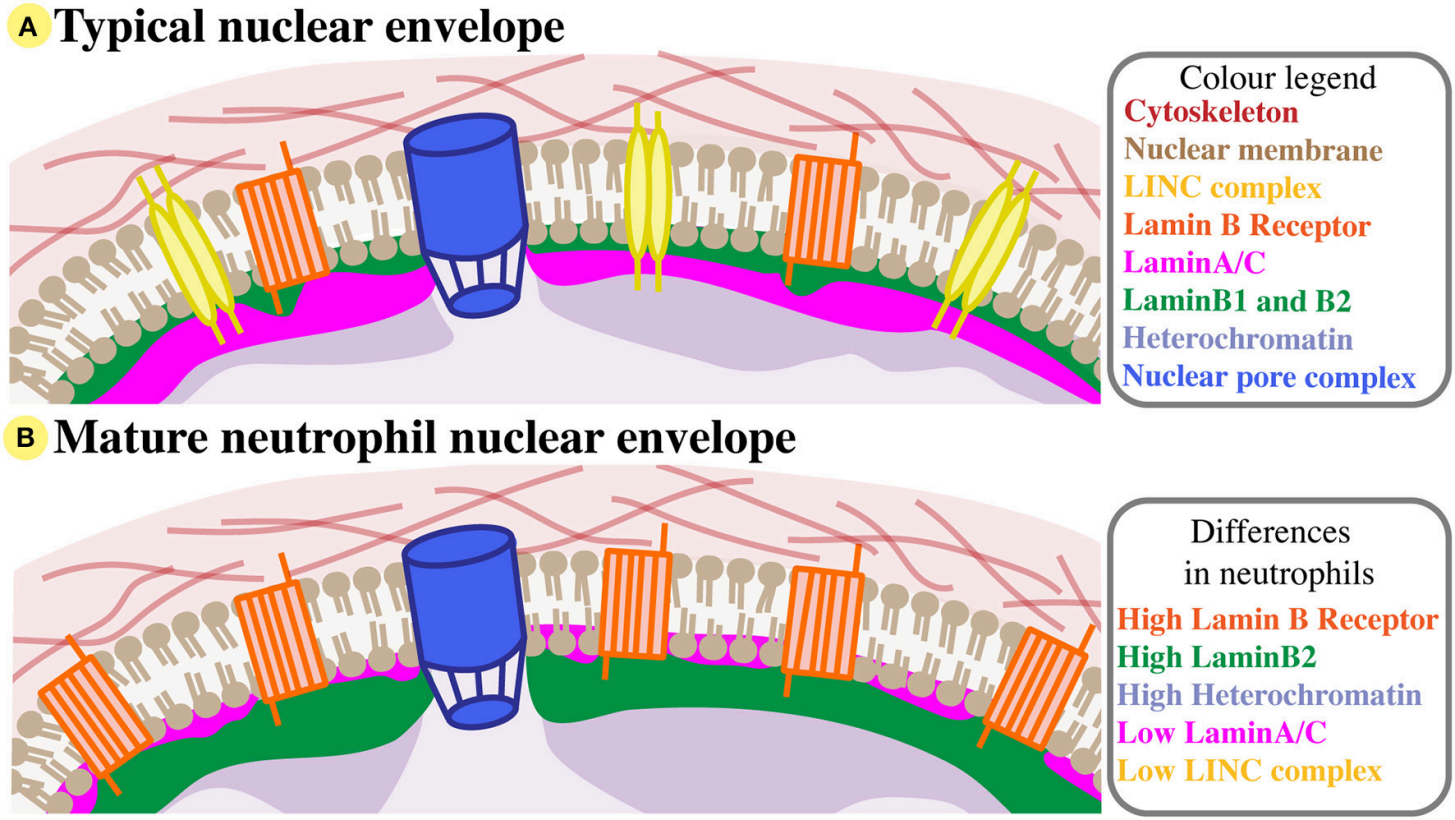
Neutrophil nuclear envelope composition. **(A)** A typical nuclear envelope comprises of the nuclear membrane bilipid layer (brown), which is embedded with membrane proteins like the LINC complex (yellow) and Lamin B Receptor (orange), and with nuclear pore complexes (blue). External to the nuclear membrane, the nuclear envelope interacts with the cytoskeleton (red). Directly beneath the inner nuclear membrane lies the nuclear lamina, a structual mesh formed of LaminA/C (pink) and B-type lamins (green). The lamina interacts with compact heterochromatin (purple). For simplicity, many nuclear membrane proteins are not shown, and LaminB2 and LaminB1 are considered together. **(B)** The nuclear envelope of mature neutrophils has very low levels of LaminA/C and LINC, but increased Lamin B receptor and peripheral heterochromatin, and relatively high levels of LaminB2.

At the nuclear boundary, the nucleus is encased by the nuclear envelope, which protects and segregates chromatin and nucleoplasm from the cytoplasm. The nuclear envelope is itself a stabilising and relatively rigid structure, and a key contributor to nuclear biomechanics. It consists of the double nuclear bilipid membrane, associated transmembrane proteins, and the nuclear lamina (Figure 1A). Spanning the nuclear double membrane, the linker of nucleoskeleton and cytoskeleton (LINC) complex is formed by envelope proteins from the Nesprin and SUN protein families (25). This LINC complex mediates nuclear-cytoskeletal coupling; the transmission of forces from the cytoplasm to nucleoplasm and vice versa (16). The LINC complex also connects with chromatin, plectin, cytoplasmic cytoskeletal elements (e.g., actin filaments, microtubules, dynein motor proteins), and nuclear lamins.

Underlying the nuclear membrane is the nuclear lamina, a mesh-like structure comprised of lamin intermediate filaments (26, 27). Lamins are tethered to the nucleoplasmic interface of the inner nuclear membrane by integral envelope proteins (28). The lamina provides mechanical support by acting as a “molecular shock absorber” (29), but also influences nuclear shape, size, flexibility and replication (30–33). Within the nucleoplasm, nuclear lamins interact with chromatin, anchoring it to the nuclear border at lamin-associated domains (34). Direct and indirect connections between lamins and histone marks alter heterochromatin distribution, hence lamins likely affect epigenetic gene regulation (35–39). The lamina is involved in nuclear-cytoskeletal coupling via its participation in the LINC complex, thus likely also plays a part in regulating mechanosensitive genes [reviewed in (40)]. Lamins have many interacting protein partners, including LINC complex components like Nesprin1α and SUN1, and inner nuclear membrane proteins such as emerin and the lamin B receptor (LBR) (41).

Nuclear lamins have highly conserved gene and protein structure (42, 43), and fall into two types: A or B. The A-type lamins are LaminA and LaminC (also written as LaminA/C), both splice variant products of the *LMNA* gene (43). LaminA is the major A-type lamin. LaminC is identical in sequence to LaminA except for the exclusion of exon 10, which truncates the C-terminus by 30 residues (44). The main B-type lamins, LaminB1, and LaminB2 are products of the *LMNB1* and *LMNB2* genes, respectively (45, 46). LaminB3 is the third B-type lamin and a splice variant of *LMNB2*, but is only expressed in germ cells (47).

A- and B-type lamins are not functionally redundant and interact with different protein partners (41). For example, LaminA/C and B-type lamins bind emerin and LBR, respectively. Recent super-resolution microscopy revealed the lamina is a heterogeneous mesh, and the A- and B- type lamin networks show no clear overlap (22, 48). This arrangement of lamin filaments could represent functionally distinct microdomains, which may explain how different lamins regulate different chromatin regions, and interact uniquely with protein partners and complexes (22, 48). Further demonstrating their capacity to perform different functions, lamin mutations result in an array of diverse nuclear phenotypes affecting nuclear shape, integrity, size and chromatin arrangement [reviewed in (40, 49)]. Since the protein composition of the nuclear envelope differs across tissues and cell types (50), there is scope for cell- and tissue-specific effects in both nuclear and cellular biomechanics.

### Neutrophils—One of the Most Mobile and Deformable Cell Types in the Body

Neutrophils are an amoeboid migratory cell type, possessing uniquely broad migratory capabilities encompassing cell speed, deformability, polarization, and directionality. To reach infection and inflammation sites first, neutrophils can have an average velocity of 19±6 μm/min *in vitro -* ~3–4-fold faster than other leukocytes like T lymphocytes (7 μm/min) and dendritic cells (2 μm/min) (51, 52), and up to 100-fold faster than mesenchymal migration of fibroblasts and invasive cancer cells (0.2–1 μm/min) (53). Moreover, neutrophils undergo rapid extravasation or transmigration, necessitating active deformation of their cellular diameter to migrate through ~1 μm endothelial channels and leave the bloodstream to access tight tissue spaces (54, 55). This is in contrast to most other cells, which cannot pass through constrictions smaller than 1.5 μm (56). Whilst the phenomenon of transmigration is well-documented and recently reviewed (10, 57), and the requirement for extreme nuclear deformability during transmigration is accepted in the literature, it has not been functionally defined.

**Figure 2:**
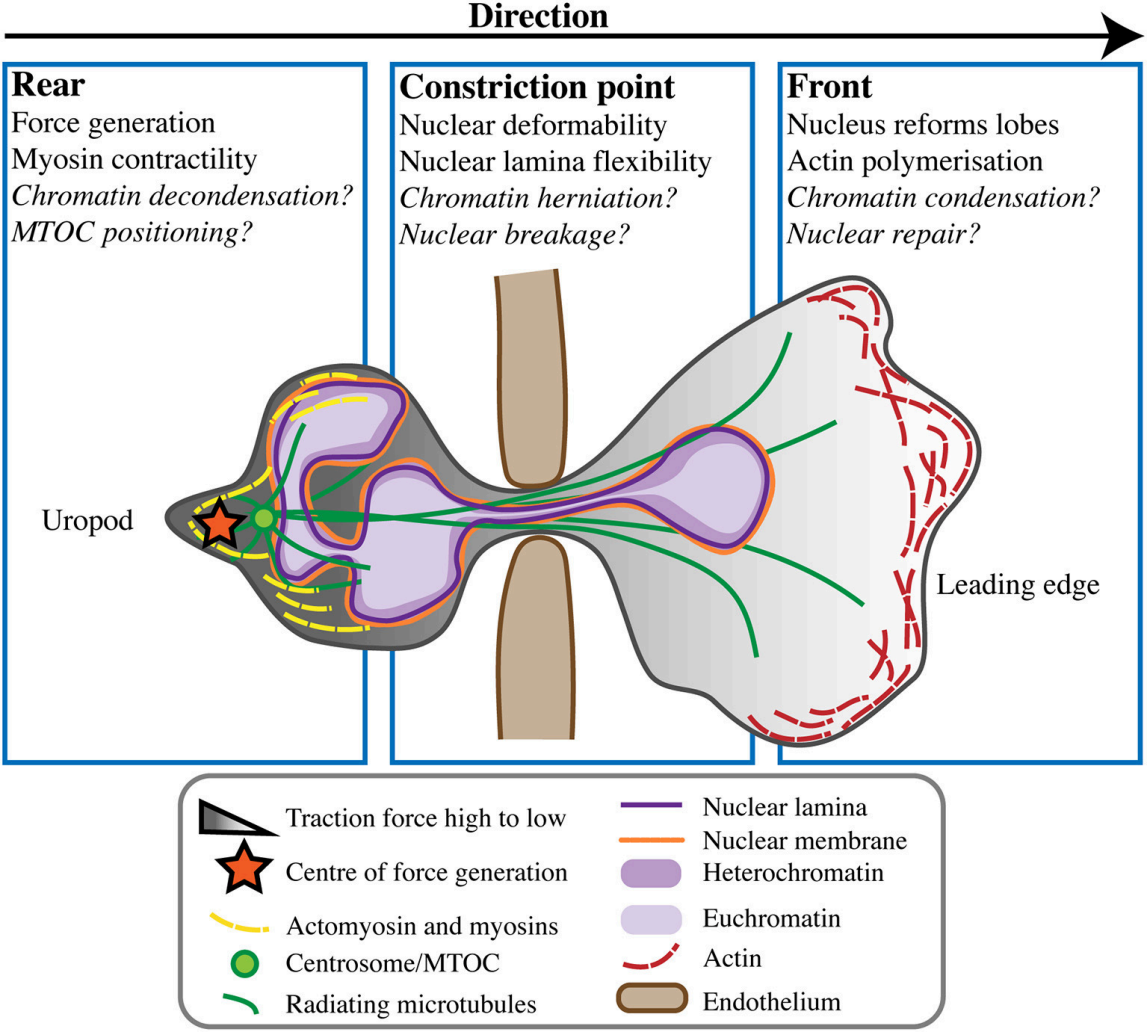
Neutrophil nuclear dynamics during transmigration. When undergoing transmigration through the endothelium (brown) neutrophils undergo extreme cellular and nuclear deformation. Different components of the cell and nucleus are believed to play roles in mechanically enabling this process, at the rear uropod, constriction point, and front of the cell toward the leading edge. Some open questions in the field remain, but the consensus is that force generation and rear myosin-mediated contractility act to push the nucleus from behind, propelling the cell forward in concert with actin polymerisation at its leading edge.

Unlike mesenchymal cell migration, amoeboid neutrophils characteristically migrate in response to traction stresses and polarised signals from the rear of the cell rather than from the front (58). This type of front-rear polarisation sees a contractile uropod at the cell rear and pseudopodia at the cell's leading edge. Contractility and force generation in the uropod causes the neutrophil to be pushed forward in a “squeezing” motion, not pulled forward [reviewed by (59, 60)] (Figure 2). Force generation behind the nucleus is believed to be necessary to push the nucleus forward, as it is a rigid intracellular “obstacle” the cell must overcome in order to move (12). However, it is unlikely that the nucleus is merely subjected to this force, rather it is a force propagator that maintains the front-rear axis and helps neutrophils move forward faster and more effectively. Using traction force microscopy in mesenchymal NIH 3T3 fibroblasts, nuclei have been shown to transmit intracellular traction forces across the cell anterior-posterior axis (3), but similar studies have not been performed for migration of amoeboid leukocytes like neutrophils.

Characteristic of amoeboid-like cells, neutrophils often display multi-directional cytoplasmic extensions and movements. Furthermore, neutrophil migration is unusual in the ability of cells to move in reverse without necessarily reversing their polarisation [(11, 61) reviewed by (10, 57)]. Reverse migration affords neutrophils the capability of not only rapidly migrating toward an immune challenge, but also of leaving it. Neutrophils can also return into the bloodstream from tissues by reverse transmigration across the endothelium. The mechanisms of reverse neutrophil migration are still unclear, but commonly involve neutrophils performing so-called “U-turns”, looping movements that occur without reversal of cellular polarity [reviewed by (60)]. Before neutrophils undergo a change in direction, the centre of force generation in the cell rear has been shown to shift, most likely in preparation for the turn (58). The nucleus may play a role in determining neutrophil directionality, via its influences on cellular mechanics and force transmission (3, 62). Interestingly, the nucleus in amoeboid leukocytes usually maintains a posterior-central position, but may translocate toward the cell's leading edge during migration due to constriction in the uropod [reviewed by (7, 51)]. As such, there could be an undescribed relationship between neutrophil nuclear position, the position of the force centre, and the ability of neutrophils to change direction.

### The Neutrophil Nucleus Is Distinct From That of Other Cell Types

**Figure 3:**
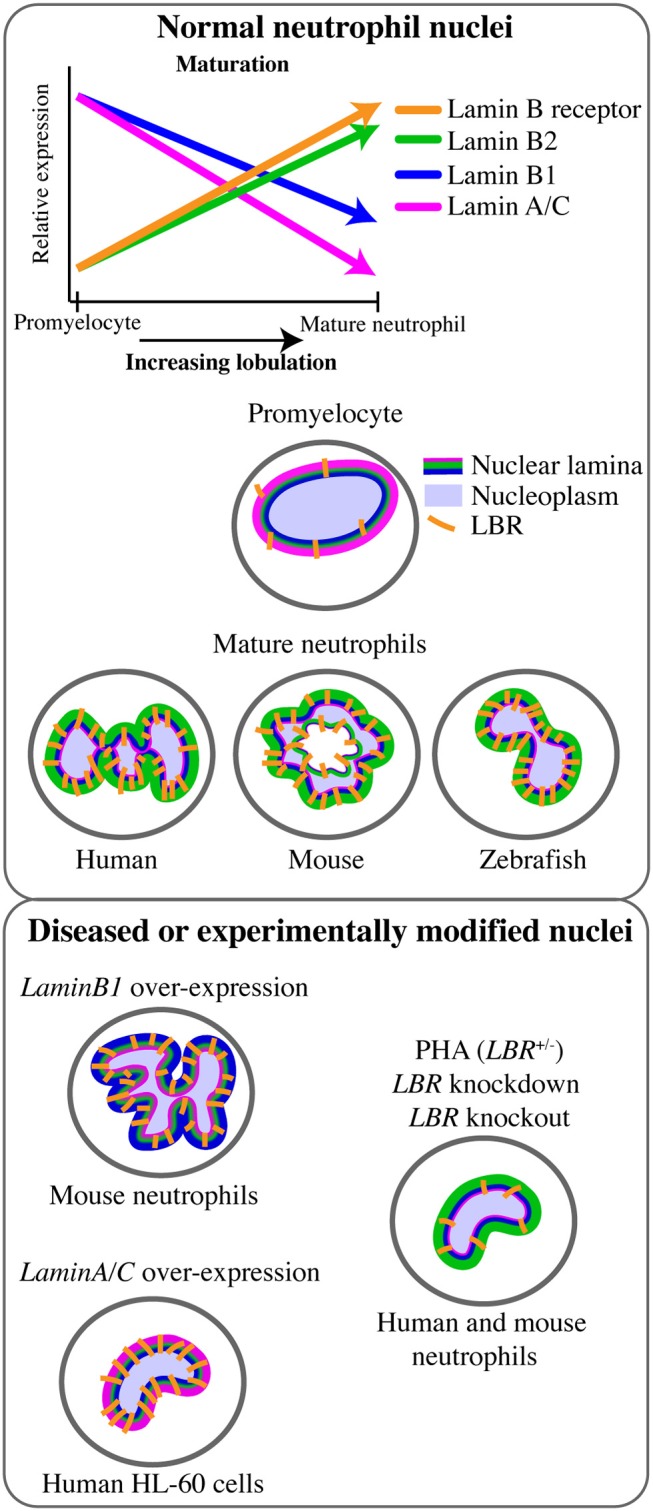
Lamin and lamin B receptor expression in neutrophils related to neutrophil nuclear morphology. Changes in the expression of lamins and the lamin B receptor (LBR) during the transition from promyelocyte to mature neutrophil occurs in tandem with increasingly lobulated nuclear shape. This multi-lobulated nuclear shape is conserved across species. The specific roles of lamin or LBR in determining nuclear morphology have been assessed functionally only in the context of LaminB1 over-expression (hyper-lobulation), LaminA over-expression (hypo-lobulation), and LBR depletion or Pelger-Huët anomaly (PHA, hypo-lobulation).

The mature neutrophil nucleus displays a unique nuclear envelope protein profile. Specifically, there is a distinct pattern of LINC, lamins, and LBR relative expression, suggesting that the neutrophil-specific combination of these nuclear components has functional importance (Figure 1B). During granulocytic differentiation, there is up-regulation of LBR, and down-regulation of LaminA/C, LaminB1, and LINC components. As LaminB2 levels remain relatively constant, LaminB2 becomes the most highly expressed lamin in mature neutrophils (Figure 3). This characteristic nuclear envelope composition is conserved across species and has been well-defined in *in vitro* studies of neutrophil-like HL-60 cell differentiation, and *ex vivo* studies of mature peripheral human blood granulocytes and mouse granulocytes (63, 64). The development of mature neutrophil nuclei that are multi-lobulated is also widely conserved across species (human, mouse and zebrafish) (64–66) (Figure 3). The parallel conservation of both distinctive nuclear envelope composition and characteristic morphology strongly suggests a dual requirement of nuclear flexibility and shape for correct neutrophil function.

## The Nucleus and Neutrophil Migration

### The Nuclear Lamina Impacts Neutrophil Migration and Cellular Plasticity

Given its central role in nuclear structure and rigidity, the nuclear lamina network is predicted to make an important contribution to neutrophil cellular biomechanics. In particular, reduced LaminA/C expression is believed to confer flexibility to the neutrophil nucleus that enables neutrophil mobility and transmigration (Table 1, Figure 2). Consistent with this, LaminA/C-deficient cells have ~50% softer nuclei and migrate more efficiently through constrictions due to increased nuclear deformability (7, 67, 80), while LaminA/C overexpression significantly impaired migration of neutrophil-like HL-60 cells through 3- and 8 μm pores in a microfluidic device. Over-expression of LaminA/C in neutrophils also induces nuclear rounding, but this morphological change is not predicted to be associated with reduced nuclear deformability (62) (Figure 3). Together, these data imply that low LaminA/C allows neutrophil nuclear flexibility, flexibility that is necessary for neutrophil migratory function.

**Table 1:** Neutrophil nuclear components and their influence on nuclear form and function.

**Nuclear component**	**Expression in mature neutrophils**	**Confirmed link to neutrophil function**	**Potential link to neutrophil function**
LaminA/C	Very low	Enables nuclear and cellular plasticity, and migration through constrictions (62, 67)	Gene expression: via epigenetic gene regulation (34)
LaminB1	Low	LaminB1 over-expression leads to nuclear hyper-lobulation (68)	Gene expression: via epigenetic gene regulation (34)
			Nuclear dynamics: influences rotation and mobility of nucleus within cytoskeletal network (69)
			Nuclear flexibility: Changes in LaminB1 expression has been associated with changes in nuclear rigidity (70, 71)
			Migration: Role in cell migration is uncharacterised except in neuronal and sperm contexts, may influence nuclear re-modelling during migration (72, 73)
LaminB2	Moderate/Low	Main component of nuclear lamina of mature neutrophils (63)	Gene expression: via epigenetic gene regulation (34)
			Migration: Role in cell migration is uncharacterised except in neuronal context (72)
Lamin B receptor	High	Reduced LBR causes neutrophil nuclei to become hypo-lobulated in Pelger-Huët Anomaly (74)	Gene expression: via epigenetic gene regulation (75)
		*LBR^+/−^* neutrophils exhibit normal phagocytosis, chemotaxis, and respiratory burst (65)	Migration: Some evidence that reduced LBR impairs neutrophil chemotaxis *in vivo* but conflicting evidence *in vitro* and *ex vivo* (62, 64)
		LBR expression influences nuclear lobulation in a dose response manner (76)
LINC proteins	Very low	Negligible expression, not expected to have explicit role in neutrophil function (63)	Nuclear flexibility and dynamics: Reduced chromatin tethering by LINC proteins likely affects neutrophil nuclear mechanics and increases deformability (63, 77)
Chromatin	High heterochromatin	Length of chromatin contacts relates to lobulated shape (78)	Nuclear flexibility and dynamics: Condensation of chromatin increases nuclear stiffness, likely influences neutrophil nuclear plasticity and stability in context of low LaminA/C, LINC (77)
		Peripheral layer of condensed heterochromatin evident in nuclei of mature neutrophil granulocytes (79)

Mechanistically, a more malleable, low LaminA/C nuclear envelope presents less resistance, providing chromatin the opportunity to potentially exert greater influence on nuclear and cellular dynamics as cells migrate (56) (Figure 2). Supporting this, softer nuclei have been shown to facilitate chromatin flow in the direction of nuclear movement (77). Reduced LaminA/C expression would also reduce the prevalence of LaminA/C-bound chromatin (39), likely affecting chromatin distribution and allowing it greater mobility within the nucleus. A recent study using micromanipulation of HeLa and MEF cells described two distinct contributions to nuclear rigidity and deformability, from LaminA/C and from chromatin (71). Cells expressing low LaminA/C showed reduced nuclear resistance such that large nuclear deformations (>3 μm) were supported, whereas reduced chromatin compaction facilitated smaller nuclear deformations (< 3 μm) but did not greatly influence larger deformations. This is consistent with neutrophil nuclei being capable of large deformations, considering their very low levels of LaminA/C and relatively high levels of compact heterochromatin (Figure 1B).

In addition, the low expression of LaminA/C in neutrophils may contribute to their relatively short life span (81). By increasing the fragility of the nuclear envelope (80), the paucity of LaminA/C likely leaves neutrophils relatively unprotected from nuclear stress and vulnerable to cell death (82). Frequent, rapid migration may contribute to increased neutrophil death as their LaminA/C-poor nuclei sustain shear stress-induced nuclear and DNA damage (83, 84). DNA damage has been associated with chromatin stiffening in yeast (85), hence the movement of chromatin material within the nucleus may become increasingly impacted as neutrophils sustain more DNA damage. Interestingly, aged neutrophils migrate faster to inflammation sites (86), potentially representing the mechanical effect of DNA damage whereby increased chromatin compaction increases deformability and mobility of the nucleus (71). Additionally, as lamin expression is implicated in ageing (87), regulation of lamins may play some part in this process.

Recent studies using microfluidic devices and nuclear localisation sequence-tagged fluorophores demonstrated that the nuclear envelope undergoes rupture and repair as fibroblasts, cancer cells, and dendritic neutrophils migrate, and that lamins are involved in this process (4, 83). Although not yet described in neutrophils, similar cycles of nuclear disruption and repair may occur as neutrophils migrate. In migrating dendritic cell leukocytes (83) and cancer cells (4) lamin-depleted blebs were observed before rupture events, suggesting a model for lamina rearrangement that allows chromatin to “leak” out as cell migrate. Super-resolution microscopy of cancer cells also identified so-called LaminA/C “scars,” where LaminA/C accumulated to repair nuclear rupture sites (4). However, it is unknown if this repair mechanism also occurs in neutrophils, which express minimal LaminA/C (Figure 2). It is possible that as a highly migratory cell type, neutrophils have a more effective nuclear repair process to survive continual nuclear rupture. Alternatively, mature neutrophils may be ineffective at nuclear repair following repeated migration, and this could contribute to their short life span.

In contrast to the high levels of LaminA/C expressed in less plastic cell types, B-type lamins are the dominant lamins in the neutrophil nuclear envelope. This suggests that increased composition of B-type lamins, specifically LaminB2, contributes to making neutrophil nuclei malleable, enhancing overall cellular plasticity. Regarding nuclear plasticity, B-type lamins have been somewhat neglected throughout the literature. A-type lamins have long been considered more important, as *LMNA* is clearly associated with many diseases, and is usually the lamin type predominantly expressed in terminally differentiated cells (88). Nonetheless, LaminB1 and LaminB2 have distinct functions and have been linked to multiple effects on nuclear biomechanics, including nuclear shape, integrity and rigidity (89).

Although *LMNB1*^Δ/Δ^ mouse embryonic fibroblasts (MEFs) have been shown to have no change in nuclear deformability compared to wild-type (90), in settings where when LaminA/C is also low, LaminB1 does appear to influence nuclear rigidity. LaminB1 over-expression has been associated with increased nuclear stiffness in leukodystrophy fibroblasts, in the absence of significantly high LaminA/C expression (70). Conversely, LaminB1 depletion resulted in nuclear stiffness in 293HEK cells that express low levels of LaminA/C(71). Given that two scenarios of low LaminA/C show LaminB1 affecting nuclear stiffness differently, there may be other proteins influencing LaminB1 effects in different cell types. No analysis of nuclear flexibility has been performed using *LMNB2*^−/−^ or *LMNB2* over-expressing nuclei. Taken together, it is not possible to completely disregard the involvement of B-type lamins in nuclear stiffness, particularly in neutrophils where LaminA/C is depleted (Table 1).

Studies investigating the role of B-type lamins in cell migration or cellular plasticity are scarce (89), and are yet to be conducted specifically in neutrophils. *LMNB2*^−/−^ mice show impaired neuronal migration to the subventricular cortex, suggesting at least to some degree that LaminB2 can affect nuclear dynamics during migration (72). *LMNB1* is also required for neuronal migration, with LaminB1 proposed to anchor the lamina such that chromatin remains sufficiently protected (91). During sperm motility, LaminB1 was shown to dynamically redistribute—indicating a potential capacity for LaminB1 to influence migration via nuclear remodelling (73). As B-type lamin deficiencies have been linked to nuclear blebbing and LaminA/C localisation (72, 92), and blebs form at lamina-depleted nuclear sites during cell migration (4), there is an argument for B-type lamin down-regulation or re-distribution as a means of mediating cell migration. Via their interaction with LBR, B-type lamins may act to redistribute chromatin as cells migrate, however this has only been shown for LaminB1 during cell senescence *in vitro* (93). There may also be an undetermined role for direct interactions between B-type lamins and heterochromatin (94).

Whilst LaminB2 is the major lamin in mature neutrophils, LaminB1 expression is down-regulated as neutrophils differentiate during granulocyte lineage progression(63) (Figure 3). When mouse bone marrow cells over-expressing *LMNB1* were used for bone marrow transplantation, granulopoiesis led to fewer neutrophils, with larger, hyper-lobulated nuclei (68)(Figure 3). This suggests LaminB1 expression influences the development, shape and function of the mature neutrophil nucleus. Sustained LaminB1 expression has been posited as necessary to maintain nuclear stiffness and integrity (71), hence down-regulation of LaminB1 in concert with low LaminA/C expression could facilitate neutrophil nuclear flexibility. Reduced LaminB1 may also indicate the neutrophil nucleus is capable of greater rotation and movement within the cytoskeletal network (69), and this increased nuclear mobility may facilitate cellular mobility. Nuclear rotation is mediated by microtubules adjacent to the nuclear envelope, and microtubules have been shown to influence neutrophil polarity and migration using live imaging in zebrafish (95). However, nuclear rotation in neutrophils has not yet been documented.

### Cytoskeletal Contribution to Neutrophil Nuclear Plasticity

It is unclear how much nuclear flexibility is due to cytoskeletal forces acting on the flexible, LaminA/C-deficient neutrophil nuclear envelope. In particular, actin networks and microtubules have been implicated in assisting nuclear constriction during migration, both *in vitro* (56) and in an *in vivo C. elegans* model (20). Thiam et al. (56) demonstrated that perinuclear actin accumulation mediated by Arp2/3 was required for required dendritic cells to deform their nuclei, and migrate effectively through narrow artificial constrictions. Perinuclear actin accumulation is also a process necessary for cancer cell nuclei to break and rupture during migration (96, 97). However, unlike cells expressing more LaminA/C and with more rigid nuclei, similar actin accumulation was not required in HL-60 neutrophils for nuclear constriction through channels as narrow as 1 μm (56).

In HL-60 cells, actin accumulation was more observable at the rear of neutrophils than at the nuclear border (56), perhaps in keeping with the contractile uropod and posterior force generation used to squeeze neutrophils forward (58, 60). Furthermore, non-muscle myosin II, an actin-binding motor protein, was necessary for neutrophil migration through 1 μm channels (56). Myosin II- mediated contractility has previously been shown to “squeeze” nuclei and is associated with rapidly migrating cells (98). Class I myosins, unconventional myosins commonly involved with cortex actin dynamics, are likely also instrumental in pushing the nucleus during neutrophil migration. Specifically, Myosin 1f is indispensable for neutrophil 3D migration*;* with microscopy demonstrating localisation of Myosin 1f at the cell rear, and *Myo1f*^−/−^ neutrophils unable to deform their nuclei through collagen matrix constrictions (99). Taken together, these data indicate a significant role for contractile myosins at the cell rear in neutrophil nuclear deformation (Figure 2).

Whilst perinuclear actin bundles appear dispensable during neutrophil migration, microtubules, another cytoskeletal element, may play crucial roles at the direct cytoskeleton-nucleus interface (Figure 2). In migrating cells, microtubules are mostly nucleated at the centrosome or main microtubule organising centre (MTOC), and radiate outwards around the nucleus. The coordination of centrosome/MTOC and nuclear positioning is important for cell migration, affecting cell polarisation, and nuclear translocation [reviewed by (100)]. MTOCs sit between neutrophil nuclear lobes in unstimulated human poly-morphonuclear leukocytes (PMNs). In PMNs that were fixed after polarised migration toward a N-Formylmethionyl-leucyl-phenylalanine (fMLP) chemoattractant gradient, 65% of MTOCs resided between nuclear lobes, and ~34% assumed a position posterior to the nucleus (101). In a similar study fixing PMNs during migration, MTOCs were shown to dynamically re-orient from the centre to the rear of the cell as neutrophils underwent polarisation (102). Live imaging of HL-60 cells moving toward an fMLP gradient further demonstrated MTOCs adjacent and posterior to nuclei (103). However, neither of these two latter studies labelled or resolved nuclear lobe structure in relation to MTOC location.

Live imaging of zebrafish neutrophils with labelled histone and tubulin showed MTOCs mainly localised in front of the nucleus, in contrast to previous *in vitro* studies (95). Anterior rather than posterior positioning may be related to the stimuli type and strength involved in migration affecting the dynamics and localisation of the MTOCs (104). This is supported by the Yoo et al. (95) observation that MTOCs move to the side of neutrophils, however the incidence of different MTOC locations was not quantified. Alternatively, the discrepancy could represent significant difference in MTOC-nucleus dynamics during 3D migration *in vivo* as opposed to 2D migration *in vitro*. Variable MTOC positioning may also relate to the activation state and immune activity of neutrophils, given that changes in MTOC positioning corresponds to immune stimulation in other leukocytes such as cytotoxic T cells (105).

The prevalent concept of rear positioning of MTOCs in neutrophils undergoing polarized migration suggests a close microtubule-nuclear envelope interaction is needed, particularly at the location of force generation, to push the nucleus forward (Figure 2). Moreover, the close proximity of neutrophil MTOCs to their nuclei suggests that microtubules act on the non-resistive nuclear envelope, and contribute to the formation of the distinct nuclear lobes in neutrophils. Consistent with this, treatment of HL-60s with paclitaxel (Taxol), a microtubule stabilising drug, resulted in induction of nuclear lobulation even in the absence of a neutrophil differentiation stimulus(106). Conversely, treatment with the microtubule inhibitor nocodazole failed to generate nuclear lobes despite retinoic-acid induced differentiation (106). Overall, it appears that cytoskeletal elements, particularly microtubules, do indeed play important roles in neutrophil nuclear deformability, positioning and lobulation. However, the localisation and dynamism of cytoskeleton-nucleus interactions awaits more precise description.

### Nuclear Shape and Neutrophil Function: Why the Lobes?

It is generally accepted that nuclear lobulation may assist neutrophil flexibility and migration, by generating less steric hindrance than round nuclei when neutrophils squeeze through the endothelium into tight tissue spaces. Yet despite this being a long-standing view, limited supporting evidence exists. Neutrophil nuclear lobes have been shown to orientate to the rear of the cell in human neutrophils fixed and examined using transmission electron microscopy (TEM) (107) and confocal microscopy (101), suggesting that nuclear lobes assume a preferential arrangement during neutrophil migration and directionality. However, apart from limited *in vitro* and *ex vivo* studies, the dynamics of these lobes during migration and their impact on migratory ability have not been explored in neutrophils migrating in a 3D environment, nor *in vivo*.

The Pelger-Huët anomaly (PHA) is a rare genetic disorder characterised by hypo-lobulated, ovoid neutrophil nuclei, as a result of autosomal dominant *LBR* mutation (74, 108) (Figure 3). As LBR expression is up-regulated during neutrophil maturation and necessary for nuclear segmentation, this has driven a widely-accepted view that this segmentation is therefore necessary for neutrophil function (109). PHA patient cells and models of PHA permit evaluation of whether or not nuclear lobulation itself improves neutrophil migration efficiency. Surprisingly, however, other than their abnormal nuclei, PHA patient neutrophils appear to exhibit normal phagocytosis, chemotaxis and a normal respiratory burst (74, 110). Studies specifically addressing lobulation and migration are limited, however, and show discordant results. The most recent *in vitro* study, using LBR knock-down by shRNA in HL60 cells, concluded that nuclear envelope composition rather than nuclear shape affected the ability to migrate through artificial pores (62). In this study LBR-depleted cells with rounded nuclei showed similar migration compared to wild-type (62). Conversely, an *in vitro* human study using primary neutrophils from five related PHA heterozygous patients showed reduced chemotaxis through small constrictions (111). *LBR-*deficient neutrophils also displayed impaired chemotaxis *ex vivo*, in studies using an *LBR*^*ic*/*ic*^ mouse model of PHA (64, 112). In addition to the discrepancy regarding the chemotactic response of *LBR*-deficient neutrophils (64, 111), evidence showing LBR does not influence the respiratory burst in human neutrophils is complicated by *LBR*^*ic*/*ic*^ mouse neutrophils showing reduced production of reactive oxygen species (64, 110). These discrepancies could represent species-specific differences as well as differences in the models (transient vs. heritable genetic alteration; cell-lines vs. primary cell culture). LBR may affect neutrophil orientation and directionality, but not migration speed, like some chemical chemotactic inhibitors (107). LBR could also influence migration via signalling pathways rather than by its effect on nuclear shape. Notably, although they can be powerful reductionist approaches, *in vitro* and *ex vivo* migration studies using artificial constrictions fails to replicate the complexity of endothelial transmigration, which may limit their power to discern effects that are more important to *in vivo* scenarios.

Other circumstantial evidence links PHA-like nuclear structure with anomalies of neutrophil function. Prompted by the observations that an acquired PHA is often seen in systemic lupus erythematosus (SLE), Singh et al. (113) examined *LBR* splicing in SLE patient neutrophils and found that aberrant mis-splicing of the *LBR* transcript was common. Furthermore, an SLE-prone mouse strain additionally carrying the *LBR*^*ic*^ mutation had an increased incidence of autoimmunity (113). This association between abnormal nuclear shape and SLE may link LBR protein function to the production of NETs, as nuclear dynamics are key for NET release (refer to following sections in this review), and SLE autoantibody production triggers excessive NETosis [reviewed by (114) and (115)]. Overall, there is a need for conclusive *in vivo* studies demonstrating how LBR-mediated nuclear lobulation variations impact on neutrophil function.

Just how PHA *LBR* mutations act structurally to actually cause the nuclear hypo-lobulation characteristic of the Pelger-Huët anomaly is unknown. Multiple hypotheses have been proposed (116), although the PHA is likely the cumulative result of several LBR functions. Firstly, LBR may structurally modulate the neutrophil nuclear envelope via its interaction with both chromatin and the neutrophil's LaminA/C-reduced nuclear lamina (a lamina that is more flexible, hence potentially more prone to lobulation). *In vitro* cell senescence studies support this, as LBR shRNA silencing caused heterochromatin detachment (93), a process linked to nuclear rounding, because it pulls the nuclear envelope inward (77). Secondly, LBR activity during mitotic nuclear re-assembly likely influences how the interphase nucleus forms, as LBR rapidly localises to the inner membrane and binds chromatin before many other nuclear envelope proteins (117). Thirdly, the sterol reductase activity of LBR (located at its C-terminal domain and hence impacted by proximal PHA mis-splicing and frame-shift mutations), may influence nuclear membrane growth and impact its shape. This hypothesis is supported by *LBR* knock-out HeLa cell *in vitro* PHA models showing impaired cholesterol metabolism (118), and *in vitro* LBR over-expression studies showing induced nuclear lobulation and excess formation of the nuclear envelope (119). A structural study using mutant forms of LBR found the N-terminus of LBR was necessary for envelope invagination, possibly via its interaction with chromatin and the lamina (119). Lastly, fluorescence recovery after photobleaching (FRAP) was recently used to examine LBR structural dynamics, finding significant differences in diffusional mobility between wild-type, N-terminal and C-terminal mutant proteins (120). *LBR* mutations may therefore affect nuclear shape by affecting LBR distribution and movement within the nuclear envelope. Furthermore, this suggests that LBR is a very dynamic protein, hence its abundant expression in neutrophils may contribute to their highly dynamic nucleus.

Not only can neutrophil nuclei be hypo-lobulated as in the Pelger-Huët anomaly, but they can also be hyper segmented. Hyper segmentation is defined as abnormal neutrophils possessing more than 5 lobes, rather than the 2-5 lobes observed in healthy cells. Neutrophil hyper segmentation can be hereditary, but cases are very rare and have been related to metabolic defects rather than defects in neutrophil structural proteins (121). Most often, hyper segmentation results from nutrient deficiency [vitamin B12, folic acid, iron (122–125)] or drug treatment, most notably G-CSF therapy (126). Hyper segmentation is long-accepted to be a maturation ‘accident' that occurs when myelocytes undergo aberrant endomitosis, resulting in DNA duplication and impaired DNA synthesis [(126), reviewed by (127)]. Given that loss of *LBR* function results in the nuclear hypo-segmentation of PHA, it is interesting that hyper segmentation accompanies an increase in *LBR* gene copy number (three gene alleles) and the consequential increased LBR protein expression (76). However, this gene dosage relationship was not absolutely linear, indicating the potential involvement of other nuclear components. There is a need for further study of the influence of hyper-lobulation on neutrophil nuclear biomechanics and function. Hyper segmentation seen in some scenarios, such as the neutrophil immune response to *Helicobacter pylori* infection (128) and tumour cells (129), suggests that hyper segmentation may either be functionally important or mark a particular functional state, and not just be a maturation mistake. Considering this, the development of a *mir-142-3p* knockout zebrafish model that displays neutrophilhyper-segmentation supplies a heritable, genetic model in which hyper-segmentation can potentially be interrogated (130).

### Chromatin Structure and Neutrophil Function

The nucleoplasmic envelope-chromatin interface likely plays a crucial part in determining neutrophil nuclear shape and function. From a gene expression perspective, lobed nuclei with specific nuclear envelope composition likely facilitate the chromatin interactions necessary for myeloid progenitors to transition into more “transcriptionally inactive” mature neutrophils. A recent study examined chromatin interactions in neutrophils (78), using a combination of Hi-C chromatin capture and mathematical modelling to compare mononuclear myeloid progenitors and polymorphonuclear neutrophils. The lobed nuclei of mature neutrophils displayed enriched long-range chromatin interactions (>3 mb), with most long-range interactions occurring in class B chromatin that is enriched for repressive epigenetic markers. Long interspersed nuclear element 1 (LINE-1) elements, ribosomal DNA, nucleoli, peri-centromeres, and centromeres were also shown to re-organise to the heterochromatic border at the nuclear lamina during neutrophil differentiation (78).

Intron retention (IR) is a form of alternate splicing that has been comprehensively assessed during granulopoiesis and implicated as a mechanism to downregulate expression of a specific gene set as neutrophils mature (68). IR regulates mRNA expression by leading to non-sense-mediated decay of IR transcripts. Interestingly, IR may directly regulate changes in neutrophil nuclear morphology as a consequence of nuclear envelope genes, including *LMNB1*, showing significantly increased IR as neutrophils terminally differentiated. Furthermore, overexpression of an intronless LaminB1 transcript not susceptible to IR-mediated downregulation resulted in impaired granulopoiesis and altered neutrophil nuclear morphology (Figure 3). Overall, this IR study contributes to the concept that neutrophil differentiation intentionally provides for changes in nuclear morphology that are closely partnered with the “switching off” of progenitor gene programs.

From a biomechanical perspective, chromatin is increasingly recognised as a structural element, affecting nuclear stiffness and mobility (77) particularly for small nuclear deformations (71). As mentioned, the flexibility of the neutrophil nuclear lamina likely means chromatin movement has greater impact on nuclear dynamics than in other cell types with more rigid nuclei. Knockdown of LINC elements in yeast suggests that low LINC expression in neutrophils, and the subsequent reduction of chromatin tethering, may also contribute to neutrophil dynamics (77). Reduced chromatin tethering increased chromatin flow, and as a result nuclei were more responsive to forces applied to the nuclear exterior by cytoskeletal microtubules. Chromatin “herniations” are also implicated in cell migration (4), although not specifically demonstrated in neutrophils, and such leakage of chromatin out of the nucleus could be facilitated by there being fewer chromatin tethers (Figure 2).

Aside from nuclear envelope composition, the lobulated nuclear shape of terminally differentiated neutrophils supports chromatin compaction at the nuclear periphery (78). This compacted chromatin forms the dense heterochromatin seen in human granulocytes by electron microscopy, which was proposed to help maintain neutrophil nuclear integrity in the absence of nuclear envelope rigidity (79). Via these various mechanisms, neutrophil chromatin arrangement and dynamics likely contribute to nuclear mobility, integrity, and lobulation, in turn influencing neutrophil migration and speed.

## The Nucleus and NETs

### Nuclear Mechanisms Underpin NET Release

It is self-evident that perturbations of the mechanisms that preserve nuclear integrity and shape must accompany the release of NETs. NETs are webs of chromatin complexed with antimicrobial proteins, released by neutrophils to ensnare and kill pathogenic microbes (13). Although considered an immune defence mechanism, excessive NET release is associated with several pathological processes, including atherosclerosis, and autoimmune disorders like systemic lupus and rheumatoid arthritis [reviewed by (114, 115)]. NETs have also been implicated in promoting cancer metastasis (131, 132).

NETosis, the process of forming NETs, occurs in response to a number of chemical and microbial stimuli. The major stages of NETosis are categorised as neutrophil stimulation, chromatin decondensation, nuclear breakdown, complexing of chromatin with neutrophil granule proteins, and chromatin release into the extracellular space. Importantly, two main forms of NETosis have been described. “Suicidal” NETosis sees the classical explosive extrusion of decondensed chromatin, involving cell membrane rupture and neutrophil cell death (13). “Vital” NETosis is characterised by more condensed chromatin being rapidly released via a vesicular exocytosis event, with the cell membrane remaining intact and neutrophils remaining viable (133, 134). Despite these descriptions of the process, much about the molecular and structural mechanisms underpinning NETosis is unknown.

The exact molecular pathways that drive NET production, as opposed to other forms of neutrophil death and DNA extrusion, are debated. Points of contention include the endpoint of lytic neutrophil death (135, 136), the involvement of specific proteins like PAD4, and the effect of different neutrophil stimuli [reviewed by (137, 138)]. Despite the controversy, nuclear mechanisms undeniably underpin NETosis, albeit mechanisms that have not been fully characterised and that likely differ between “suicidal” and “vital” NETosis.

**Figure 4:**
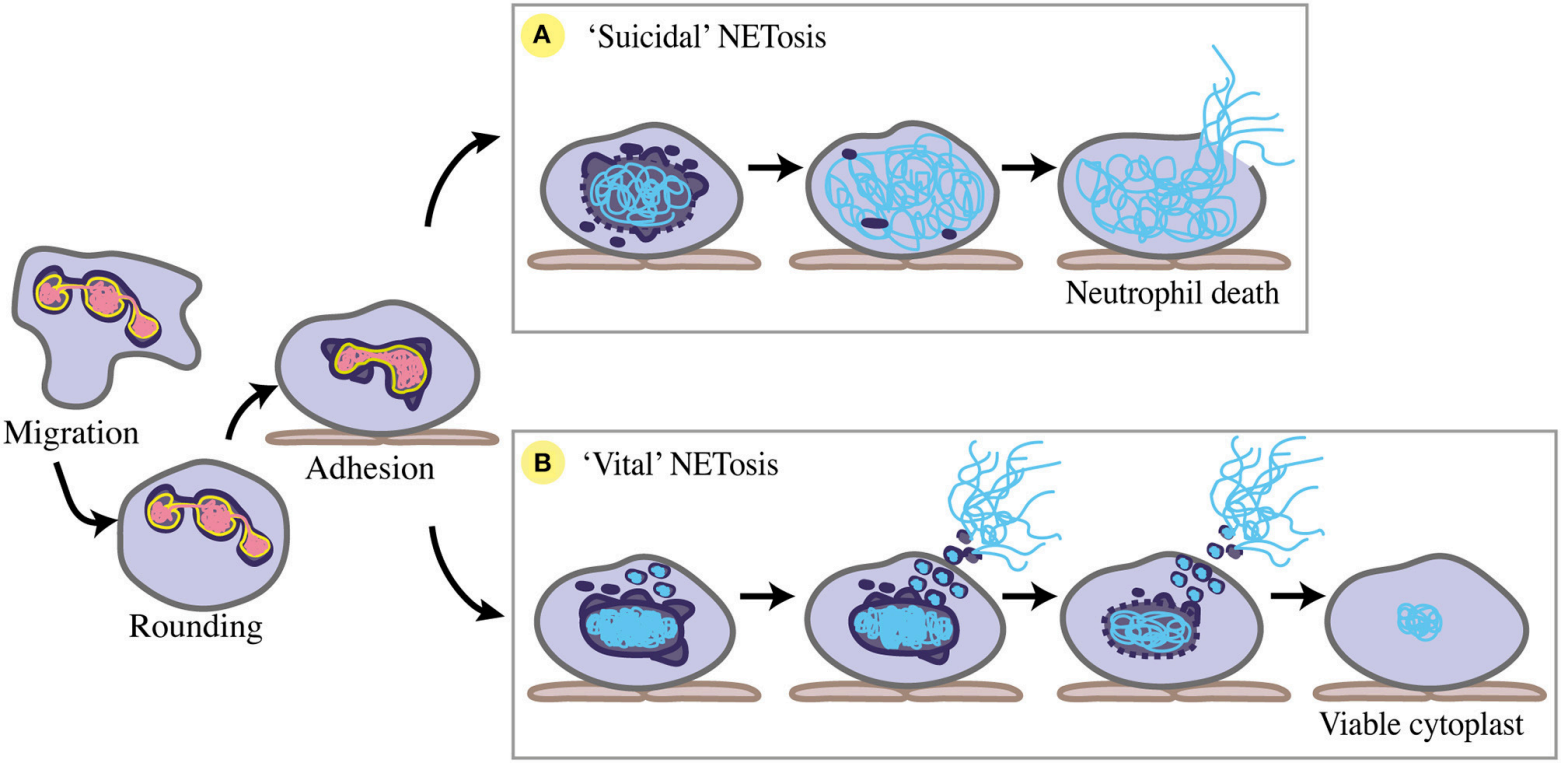
Nuclear changes during NETosis. Prior to NETosis, a neutrophil carries a typical multi-lobulated nuclear envelope (dark purple), with distinct euchromatin (pink) and heterochromatin (yellow). Upon stimulation toward NETosis, the neutrophil rounds up, then adheres to the endothelium (brown) where chemotaxis is arrested, and the nuclear envelope begins to dilate and round. **(A)** For suicidal NETosis, nuclear vesicular budding begins early after stimulation, leading to nuclear envelope breakdown and chromatin decondensation. Decondensed chromatin (blue) swells and is no longer distinguishable as eu- or hetero-chromatin. The nuclear envelope completely breaks down, and decondensed chromatin fills the cytoplasm. NET release occurs via cell lysis as cell membrane (grey) ruptures, ultimately resulting in neutrophil cell death. **(B)** For ‘vital' NETosis, initially the nuclear envelope remains intact, with some release of nuclear vesicles containing DNA material. Nuclear chromatin condenses, and is no longer eu- or hetero-chromatin. DNA-containing vesicles fuse with the cell membrane, and NETs are released as these vesicles lyse in the extracellular space. As NET release continues via nuclear budding, nuclear chromatin decondenses and detaches, and the nuclear envelope breaks down. Completion of nuclear breakdown sees the post-NETosis neutrophil remain viable and functional as a cytoplast with some DNA material remaining in the cytoplasm.

For chromatin webs to be released, the neutrophil nucleus must at least partially disassemble. Nuclear envelope breakdown during “suicidal” NETosis was shown using transmission electron microscopy (TEM) of human neutrophils stimulated by PMA *in vitro* (139). Pilsczek et al. (134) have since conducted an extensive descriptive TEM study of the nuclear stages involved in “vital” NETosis *in vitro*, supported by the *in vivo* studies of Yipp et al. (133). Collectively, several nuclear changes have been identified during NETosis, including nuclear rounding and blebbing, chromatin condensation and decondensation, nuclear rupture, and nuclear disassembly (Figure 4). Due to its similarities to mitosis, NETosis has been considered a form of commandeered or reconfigured cell division (140), rather than modified apoptosis or necrosis. However, many aspects of nuclear dynamics during NETosis are yet to be specifically defined, including the precise participation of nuclear envelope components like lamins and LBR (Table 2).

**Table 2:** Potential roles for chromatin and nuclear envelope components in the stages of NETosis.

**NETosis event**	**Hypothesised roles of nuclear components**
Nuclear rounding/de-lobulation	Nuclear envelope hypo-lobulation may be due to LBR down-regulation (74)
	Lamin concentration and distribution may influence nuclear size and shape (31)
Nuclear blebbing	Down-regulation or redistribution of lamins may result in blebbing (72)
	LBR may influence nuclear membrane invagination (119)
	Blebs may represent mechanism for chromatin to initiate nuclear rupture (4)
Nuclear translocation	Lamins may mediate redistribution of nuclear pore complexes, affecting nuclear transport during NETosis (22)
Chromatin detachment	Detachment possibly mediated by paucity of chromatin-tethering LINC and emerin, and LBR may be a significant chromatin-tethering component in their absence (134)
	Interactions of lamins and LBR with chromatin and chromatin binding proteins may influence detachment (39, 41, 92)
	The prevalence of peripheral heterochromatin and mitosis-like histone modifications in mature neutrophil nuclei may affect chromatin dynamics (79, 140)
Chromatin condensation and decondensation	Lamins and LBR interact with chromatin and chromatin binding proteins, may affect epigenetic modifications and chromatin condensation state (39, 41, 92)
	Condensation state of chromatin affects nuclear rigidity, hence likely affects nuclear dynamics during NETosis (77)
	Reduced LaminA/C reduces nuclear envelope resistance, chromatin may exert more mechanical force during NETosis (56)
	The prevalence of peripheral heterochromatin and mitosis-like histone modifications in mature neutrophil nuclei may affect chromatin dynamics (79, 140)
	Chromatin decondensation and swelling is a key mechanical force for nuclear and cellular rupture during lytic NET release (141)
Nuclear breakdown/rupture	Nuclear disassembly may be regulated by lamins and LBR, similar to disassembly during mitosis (117, 134, 140)
	Low LaminA/C and decondensed chromatin reduce nuclear stiffness, likely resulting in a nucleus more susceptible to rupture for NET release (77, 80)
	Mechanical force exerted by chromatin on the nuclear envelope likely assists nuclear rupture (4)

### Nuclear De-lobulation Precedes NET Formation

Prior to both “suicidal” or “vital” NETosis, neutrophil nuclei undergo a transition from multi-lobulated to rounded. De-lobulation precedes both chemically-induced and *S. aureus*-induced NET release *in vitro* (134, 141–143). Nuclear de-lobulation may indicate that LBR is down-regulated, and that heterochromatin detachment has begun (77, 93). In addition to nuclear rounding, the inner and outer nuclear membranes detach from one another, undergoing “blebbing” as the perinuclear space dilates (134, 139). This suggests structural changes to the nuclear lamina, as lamin deficiencies often result in abnormal nuclear blebbing (72). Moreover, modulation of LBR levels may mediate envelope dilation as its overexpression/overproduction influences nuclear membrane structure, causing invaginations (119). In cell migration, nuclear membrane detachment and lamina-depleted blebs presumably facilitate nuclear rupture (4). A similar process could occur during NET formation.

### Chromatin Condensation and Decondensation During NETosis

As NETosis initiates in neutrophils, chromatin remains organised distinctly into hetero- and euchromatin. However, the condensation state of chromatin changes dramatically during both “suicidal” and “vital” NET formation. The low LaminA/C content of the neutrophil nuclear lamina, which makes for a less stiff nuclear envelope, presumably allows the condensation state of chromatin to exert considerable influence on neutrophil nuclear dynamics and shape (56). Interestingly, comparison of NETs stimulated by different chemical and microbial stimuli sees differences in chromatin decondensation kinetics, suggesting different regulation of chromatin decondensation affects the onset and speed of NET responses (141, 144, 145).

As “vital” NETosis progresses, the nucleus becomes increasingly rounded and chromatin is condensed (134). Interestingly, chromatin condensation has not yet been noted in the production of lytic NETs. This either represents a key difference between the forms of NETosis (146), or a rapid step that has been missed in the absence of comprehensive *in vivo* imaging. More rigid, condensed chromatin structure is associated with increased nuclear stiffness and force transmission by nucleo-cytoskeletal coupling (147). As neutrophils exhibit reduced expression of LINC components (63), nucleo-cytoskeletal coupling may play a lesser role in neutrophil chromatin condensation (56). Alternately, LBR, one tethering protein that is still highly expressed in neutrophils, could be mechanically important during NET release—considering the absence of significant “input” from other tethering proteins. As total lamin concentration has been linked to nuclear size (31), the distribution of lamins may also play a role in this nuclear compaction phase of NETosis.

Nuclear chromatin decondensation is a key step preceding nuclear breakdown during “suicidal” and “vital” NET release (133, 139, 148), and is evidenced by nuclear swelling of NETosing neutrophils, both *in vitro* and *in vivo* (133, 141). During “suicidal” NETosis of primary neutrophils *in vitro*, chromatin decondensation and swelling has been found to be a dominant contributor of mechanical force necessary for the lytic release of NETs (141). Neubert et al. (141) used a combination of single cell confocal, simulated emission depletion, and atomic force microscopy to monitor chromatin swelling and describe three biophysical phases of “suicidal” NETosis. Firstly, stimulated neutrophils round their nuclei (as previously described) in conjunction with performing active biochemical processes, primarily enzymatic activity and histone modifications. Chromatin decondensation leads to swelling and a switch to the second phase, whereby NETosis becomes a passive, ATP-independent process that is dictated by the outward pressure exerted by swelling chromatin. The switch from the first and second phase includes a so-called “point of no return,” when chromatin swelling becomes so great that it cannot be reversed. Chromatin expands such that it breaks out of the nuclear envelope, and continues swelling to fill the cytoplasm. In the third and final phase, expanded chromatin ruptures the cell membrane, releasing the NET. Intriguingly, in a majority of cases a localised region of the nuclear border is in closer proximity to the cell membrane than elsewhere. Chromatin pressure becomes concentrated in this region, and this concentration allows some prediction of the final rupture point through which the NET exits through the cell membrane. Supporting the concept that nuclear positioning impacts the directionality of cell membrane rupture and final NET release, NET release from neutrophils with centrally located nuclei occurred at random.

Histone modifications occurring in the early stages of NETosis see a reduction in positive charge exerted on negatively charged DNA, allowing it to swell and decondense (11, 141). PAD4-mediated histone H3 hyper-citrullination, considered a requirement for NET release, is proposed as a mechanism for this chromatin decondensation (148, 149). Rearrangement of chromatin architecture during NETosis may be directly or indirectly facilitated by nuclear envelope proteins like lamins and LBR, as these proteins interact with histone marks and histone-binding protein partners, and their distribution in the nuclear envelope has been shown to affect heterochromatin localisation in other biological contexts (39, 93). Of most interest, LBR has been shown to bind histone H3 (75) but its role in citrullination has not yet been investigated. DNA elements that are localised to the nuclear periphery during neutrophil maturation may also have additional properties that that assist chromatin re-modelling, and functionally prepare neutrophils for NET release to occur (78).

### Nuclear Translocation of Neutrophil Proteins for NET Production

At the initiation of NETosis, there is nuclear translocation of cytoplasmic granule proteins such as myeloperoxidase, and proteases including neutrophil elastase and SerpinB1. The entry of these proteins into the nucleus is believed to assist chromatin decondensation by complexing with chromatin for NET formation but ultimately their incorporation into the NET contributes to its antimicrobial activity (143, 150–152). LaminA/C has been implicated in the clustering and distribution of nuclear pore complexes (NPCs) in the nuclear envelope (22). As NPCs control large molecule transport into and out of the nucleus, the lamina could potentially influence the trafficking of these proteins during NETosis. This is supported by evidence that NPCs remain intact until late NETosis (134). Additionally, from recent plant studies, links are emerging between NPC rearrangement, immune signalling, and cell death (153). Lamins could influence general neutrophil signalling and cell death by regulating NPC localisation. However, NPC distribution has not yet been documented in studies of neutrophils or NETs.

### Chromatin Detachment From the Nuclear Envelope

“Vital” NETosis of human neutrophils exposed to *S. aureus* showed nuclear envelope “tubules” detached from the chromatin before nuclear breakdown (134). This phenomenon, envelope detachment from the heterochromatin, resembles what occurs in laminopathies (specifically LaminA/C deficiency) or defects in nuclear proteins such as emerin that are involved in chromatin tethering (134, 154). Intriguingly, heterochromatin detachment has also been described when LBR is down-regulated (93). Whilst chromatin detachment has so far only been detailed during “vital” NETosis, these TEM images suggest that lamins and other nuclear envelope proteins like LBR play a role in regulating the de-tethering of chromatin for NET formation.

More specifically, chromatin detachment suggests the paucity of LaminA/C and chromatin-tethering proteins in neutrophils allows their chromatin to be loosely anchored to the nuclear envelope, and more readily decondensed and released during NETosis (134). This hypothesis is further supported by reduced chromatin tethering being shown to reduce nuclear stiffness and chromatin viscosity (77). Hence detached chromatin likely means the neutrophil nucleus is less resistant to mechanical force, and can more easily re-model and rupture for NET release. This hypothesis could be tested by the over-expression of lamins or other chromatin-binding proteins like emerin and LBR, which would potentially inhibit or impair NET release, shedding light on the involvement of these structural nuclear proteins in NETosis.

### Vesicular Budding, Nuclear Breakdown, and Capacity for Repair

*in vitro*, TEM has clearly displayed the nucleus dissipating during “suicidal” and “vital” NET release, via vesicular budding of the nuclear envelope (134, 139). For “suicidal” NETosis, the nuclear envelope disintegrates prior to decondensed chromatin filling the cytoplasm, mixing with granule components, and lytic release as a NET (139). Interestingly, *in vitro* immuno-staining has shown that the nuclear lamina starts to break before disintegration of the entire nucleus begins, rupturing early as increasingly decondensed chromatin swells and can no longer be contained by the lamina (141). For “vital” NETosis, exocytic release of NETs can occur before nuclear breakdown initiates, with complete breakdown occurring later (134) (Figure 4). *In vivo* imaging of “vital” NETosis in a mouse skin *S. aureus* infection model identified neutrophils displaying one of three nuclear phenotypes: normal, diffuse, or absent (133). The “diffuse” nuclear phenotype was considered concordant with neutrophils undergoing nuclear envelope breakdown and has also been observed *in vitro* (142).

Vesicular budding of the nuclear envelope may indicate roles for lamins and LBR in nuclear breakdown during NETosis. Although the presence of lamins in these vesicles has not been determined, LBR has been shown to be present by *in vitro* immuno-staining (139). The involvement of lamins and LBR in nuclear disassembly during cell division may provide further clues as to how these proteins are involved in forming NETs (6, 117, 155). Indeed, TEM images suggest that nuclear disassembly in NETosing cells occurs analogously to that of prometaphase mitosis (134). Cell cycle and mitotic proteins have also been implicated in PMA- and *C. albicans* induced NETosis, including the phosphorylation of LaminA/C (140). This strengthens the concept of NETosis as “modified” or “hi-jacked” cell division. Throughout this, the low expression of LaminA/C and LINC proteins in the neutrophil nuclear envelope likely assists breakdown, by increasing nuclear fragility and susceptibility to chromatin-exerted pressure.

Completion of nuclear breakdown can lead to different NETosis endpoints (Figure 4). “Suicidal” NETosis ends with the release of highly decondensed chromatin into the cytoplasm, where it complexes with additional neutrophil proteins (released from cytoplasmic granules) before explosive extrusion out of the neutrophil (139). “Vital” NETosis proceeds with DNA-containing vesicles that have budded off the nucleus fusing with vesicles containing granule proteins, before fusing with the cell membrane to enter the extracellular space, where they lyse and form NETs (134). These enucleate neutrophils remain viable as cytoplasts that retain functionality *in vitro* and *in vivo*, still performing phagocytosis, chemotaxis, and adherence (133, 156). Furthermore, retention of cytoplasmic DNA by some anuclear, viable neutrophils post-NETosis *in vitro* suggests that not all DNA material need be extruded to form NETs (134).

Neutrophils undergoing “suicidal” NETosis likely die due to irreparable nuclear envelope and DNA damage as well as cell membrane rupture (13, 83, 139). Regarding “vital” NETosis, the presence of anuclear, viable neutrophils is supported by the survival of cells lacking nuclear lamina (157), and may mean neutrophils have removed or “ejected” damaged nuclei that would otherwise induce cell death (83). Moreover, the protective activity of lamins at the nuclear envelope (82) could determine whether or not a NETosis event results in neutrophil death or viability. Experimental down-regulation or up-regulation of lamins may therefore provide additional insight into the differences between “suicidal” and “vital” NETosis.

### Nucleo-Cytoskeletal Interactions During NETosis

Unsurprisingly, cytoskeletal elements have also been shown to influence the nuclear and cellular mechanics required for NET formation. Changes in tubulin and actin polymerisation accompany histone citrullination and nuclear breakdown during NETosis (158). Microtubule dynamics and the movement of MTOCs during NETosis mirror those of mitosis (140), however microtubule rearrangement occurs prior to extreme chromatin decondensation, hence microtubules are unlikely to be crucial for moving genetic material and rather involved in the active initiation phase of NETosis (141). Similarly, actin dynamics appear more important in the early stages of NETosis, as chemical inhibition of actin polymerisation late in NETosis does not prevent NET release (141). Actin polymerisation is modulated by myeloperoxidase and neutrophil elastase, with a myeloperoxidase-containing granule protein complex called the “azurosome” mediating neutrophil elastase nuclear translocation that ultimately inhibits actin extension into the cytoplasm (151). Collectively, it appears that cytoskeletal actin rearrangement, although required for NETosis, is stalled and discontinued as neutrophils prepare increasingly decondensed chromatin for NET release (141). Notably, cytoskeletal dynamics have been investigated in detail only in terms of lytic “suicidal” NETosis, thus there remains potential for alternative cytoskeletal mechanisms to be involved in exocytic “vital” NETosis.

### Looking Forward: The Study of Neutrophil Nuclei and NETs

In the last decade, the field of neutrophil biology has made growing use of microscopy techniques such as confocal laser scanning and spinning disk microscopy, transmission and scanning electron microscopy, super-resolution imaging [like simulated emission depletion microscopy (STED)], and atomic force microscopy (AFM) to visualise and mechanically interrogate neutrophils and NET production *in vitro* (141, 159). Moving forward, there is scope for higher resolution 4D imaging of neutrophil nuclei in complex 3D environments, especially *in vivo*. This would capture nuclear plasticity and dynamics during migration and NET release, allowing for greater descriptive and mechanistic investigation. Tools for *in vivo* live imaging approaches include conventional confocal microscopies (11, 133, 160), but also emerging higher-resolution 4D techniques like multiphoton intravital imaging [reviewed by (24)], single plane illumination microscopy (SPIM, also known as lightsheet fluorescence microscopy, LSFM) (161) and lattice lightsheet microscopy [(162, 163), reviewed by (23)] enhanced by the application of adaptive optics (164). Lightsheet microscopies in particular allow far greater spatiotemporal resolution, with cellular events able to be captured in almost real-time. In addition to microscopy advances, microfluidic devices are gaining popularity for controlled *in vitro* and *ex vivo* studies of leukocyte biology [reviewed by (21)]. Microfluidics will likely be a powerful tool for the ongoing study of neutrophil nuclei, particularly the migration of neutrophils with labelled nuclei, and aspects of NETosis (165).

## Conclusion

The nuclear envelope is a key contributor to nuclear plasticity, with envelope proteins such as lamins and Lbr affecting nuclear shape, flexibility and chromatin dynamics. The distinctive nuclear envelope composition of neutrophils is believed to impact the function of their multi-lobulated nuclei, particularly during migration and NETosis—processes which require nuclear deformation and re-modelling. However, despite the description of neutrophil nuclear shape, flexibility and chromatin composition, the functional roles of specific nuclear components, namely B-type lamins and LBR, warrant further investigation. Similarly, those moieties interacting with either side of the nuclear envelope, specific cytoskeletal elements and chromatin elements, require further study. From a cellular biology viewpoint, neutrophils, with their extraordinary cellular plasticity and unique nuclei, provide a paradigmatic opportunity to explore the relationship between nuclear structure and cellular function. From a leukocyte perspective, establishing the contribution of the nucleus to neutrophil migration and NETosis will undoubtedly provide valuable information on neutrophil behaviour during immune responses, particularly inflammatory disorders.

## Author Contributions

HM wrote the paper. GL and MK contributed to writing and critically revised the paper.

### Conflict of Interest Statement

The authors declare that the research was conducted in the absence of any commercial or financial relationships that could be construed as a potential conflict of interest.
